# Combining Ability and Hybrid Performance of Cowpea Landraces Under Semiarid Conditions

**DOI:** 10.3390/plants15203145

**Published:** 2026-10-14

**Authors:** Maria Joelma da Silva, Samandra Silva de Lima, Mateus de Souza Silva, Vitor Emanuel Conrado da Silva, Taís Borges da Silva, Rubens Rangel Rolim, João Henrique Constantino Sales Silva, Helder Farias Pereira de Araújo, Edna Ursulino Alves, Lenyneves Duarte Alvino de Araújo, Naysa Flávia Ferreira do Nascimento

**Affiliations:** 1 Department of Plant Science and Environmental Sciences, Center for Agrarian Sciences, Federal University of Paraiba, Campus II, Areia 58397-000, PB, Brazil; joelma_mjs@outlook.com (M.J.d.S.); limasamandra@hotmail.com (S.S.d.L.); mateussouza7474@gmail.com (M.d.S.S.); vitoremanuelagro@gmail.com (V.E.C.d.S.); thaysborgesb55@gmail.com (T.B.d.S.); ursulinoalves@hotmail.com (E.U.A.); naysa.flavia@academico.ufpb.br (N.F.F.d.N.); 2 National Institute of Colonization and Agrarian Reform, João Pessoa 58031-000, PB, Brazil; rubensrangel.rrr@gmail.com; 3 Department of Biosciences, Center for Agrarian Sciences, Federal University of Paraiba, Campus II, Areia 58397-000, PB, Brazil; helder@cca.ufpb.br (H.F.P.d.A.); lenyneves@academico.ufpb.br (L.D.A.d.A.)

**Keywords:** Caatinga, diallel analysis, food security, participatory breeding, *Vigna unguiculata*

## Abstract

Cowpea (*Vigna unguiculata* (L.) Walp.) plays a key role in food security and income generation in semiarid regions, particularly for smallholder farming systems in the Brazilian semiarid. However, productivity remains low because of environmental limitations, genetic constraints, and traditional crop management practices. We evaluated diallel crosses among cowpea landraces to identify superior parents and cross combinations in terms of earliness and grain yield under semiarid conditions. Six landrace accessions, previously selected through participatory prospecting for adaptability and yield stability, were crossed in a complete diallel design to produce 15 F_1_ hybrids and their reciprocal crosses. The hybrids were evaluated under field conditions in the semiarid region during the 2023 growing season using a randomized complete block design with ten replications. The evaluations were conducted according to each genotype’s developmental stage and immature pod formation. Early-maturing varieties were harvested at 60 days, intermediate and late varieties between 65 and 75 days. At physiological maturity, early and intermediate genotypes were harvested at 80 days, while late genotypes were harvested between 85 and 90 days. General combining ability (GCA) and specific combining ability (SCA) were estimated using Griffing’s Method I (fixed-effects model), and treatment means were compared using the Scott–Knott test at the 5% significance level. Significant GCA and SCA effects (*p* < 0.05) were detected for 12 of the 14 evaluated traits, whereas reciprocal effects were significant for seven traits. The hybrid combinations L1 × L4, L1 × L5, and L4 × L5, and their reciprocal crosses, exhibited superior agronomic performance, highlighting their potential as valuable genetic resources for cowpea breeding under semiarid environments.

## 1. Introduction

Semiarid regions encompass vast areas inhabited by millions of people and are characterized by high levels of socioeconomic and environmental vulnerability [1]. Similar challenges occur across the drylands of Africa, Asia, and Latin America, where water scarcity, soil degradation, and the effects of climate change interact to intensify the vulnerability of rural populations and threaten the sustainability of agricultural production systems [2].

These constraints highlight the need for crop species adapted to water-limited environments to ensure the sustainability of local production systems. Among the crops cultivated by smallholder farmers in the semiarid region, cowpea (*Vigna unguiculata* (L.) Walp) is particularly important legume species because of its broad adaptation to tropical and subtropical regions of the world, as well as its tolerance to water deficit and suitability for low-input production systems [3,4,5]. In addition to its agronomic adaptability, the black-eyed pea is an important source of plant protein and is of nutritional, economic, and social significance, especially in resource-limited environments [6,7,8]. Breeding programs at IITA and national institutions have developed and made available cultivars with higher yields, stress tolerance, nutritional quality, and adaptation to low-input farming systems [3]. However, the high climatic variability in semiarid regions still poses challenges to the adaptation of these cultivars, highlighting the need to expand the available genetic base and develop germplasm better adapted to these conditions [9,10].

This challenge is equally evident in Brazil, where only 71 cowpea cultivars are currently registered in the National Register of Cultivars (RNC) [11], of which only 12 have been released during the last decade. Despite approximately 1.25 million hectares cultivated nationwide, the average grain yield was only 478 kg ha^−1^ during the 2024/2025 growing season [12]. In the Cariri region of Paraíba, cowpeas are grown primarily on small plots by family farmers, usually using rain-fed farming systems and in intercropping with corn and other annual crops; they are an important crop for food security and income generation [13,14]. Grain yield in the Paraíba semi-arid region ranges from 381 to 1147 kg ha^−1^ depending on the variety [13]. The main challenges facing this crop in the region are irregular rainfall and periods of drought, as well as low adoption of technologies, limitations in soil fertility and management, the occurrence of pests and diseases, and, in irrigated areas, risks of salinity [14,15,16].

This diversity increases the likelihood of genetic complementarity among the parents, favoring both additive and non-additive genetic effects and, consequently, enhancing general and specific combining ability [17,18]. Therefore, these landraces represent valuable genetic resources for developing hybrids and segregating populations with superior agronomic performance.

Although numerous studies have evaluated commercial cultivars and advanced breeding lines for grain yield, adaptation, and genetic gain, the potential of landraces as parental lines in hybridization programs remains largely underexplored [19]. Some studies include local varieties among the materials evaluated, but their potential as parental sources of alleles of interest is not yet the central focus of this research. In this context, information on the combinatorial potential of local varieties conserved by farmers remains limited, especially when used in diallel crosses to identify promising combinations under semiarid conditions.

In this context, hybridization represents a strategic approach to cowpea breeding, as it allows for the combination of favorable alleles from complementary parents to develop lines with high yield potential and good adaptability, while also increasing the efficiency of selection [20,21]. Among the available breeding approaches, diallel analysis is widely used to estimate general and specific combining ability, providing valuable insights into the relative contributions of additive and non-additive genetic effects controlling agronomic traits. These estimates facilitate the identification of superior parental genotypes, the selection of promising hybrid combinations, and the design of effective breeding strategies [21].

Given this scenario, exploiting the genetic variability present in cowpea landraces represents a promising strategy for developing cultivars adapted to semiarid environments, thereby contributing to food security and the sustainability of agricultural production systems. Accordingly, we evaluated the combining ability and agronomic performance of hybrids obtained from diallel crosses among cowpea landrace. Our objective was to identify superior parents and hybrid combinations with favorable grain yield performance, with the aim of contributing to the selection of materials with greater performance potential under semiarid conditions.

## 2. Results

Analysis of variance (Table 1) revealed significant treatment effects (*p* < 0.01) for all evaluated traits. General combining ability (GCA) showed significant effects (*p* < 0.05) for thirteen of the evaluated traits, with the exception of dry grain yield. Specific combining ability (SCA) was significant for twelve of the evaluated traits, except for the number of dry grains and the weight of 100 dry grains. Significant reciprocal effects were detected for six of the 14 traits evaluated (42.8%), namely: stem diameter, plant height, central leaflet length, central leaflet width, green pod length, and weight of 100 dry seeds.

The ratio between the mean squares for GCA and SCA varied among traits, indicating differences in the relative contributions of additive effects and dominance deviations to trait expression (Table 1). Values greater than one were observed for central leaflet width, green pod length, and 100-green-grain and 100-dry-grain weights, indicating the predominance of additive gene effects in the control of these traits. For stem diameter, leaflet area, plant height, central leaflet length, number of pods per plant, number of green grains, green grain yield, dry pod length, and dry grain yield, non-additive gene effects were predominant.

The genetic correlation coefficients among the evaluated traits showed both positive and negative values (Table S1—Supplementary Materials), demonstrating that the evaluated traits exhibited both positive and negative genetic correlations. The highest correlation was observed between green pod length and the number of green seeds (*r* = 0.77), followed by the correlation between dry pod length and the number of dry seeds (*r* = 0.75) and between leaf area and central leaflet length (*r* = 0.73). For dry grain yield, the highest correlation was observed with 100-dry-grain weight (*r* = 0.70), indicating a positive genetic relationship between these traits. A positive correlation was also observed between green pod length and the number of dry seeds (*r* = 0.70).

Based on the mean values grouped by the Scott–Knott test at the 5% significance level, considerable variation was observed among the evaluated parents and hybrid combinations (Table S2—Supplementary Materials). Among the parents, L1 and L3 showed superior performance for stem diameter, leaflet area, plant height, central leaflet length, central leaflet width, green pod length, number of green grains, 100-green-grain weight, green grain yield, dry pod length, 100-dry-grain weight, and dry grain yield. Among the hybrid combinations, L1 × L4, L1 × L5, and L4 × L5, exhibited superior performance for stem diameter (0.98 to 1.61 cm), leaflet area (162.68 to 298.28 cm^2^), number of green grains (10.40 to 16.33), green grain yield (1731.94 to 4640.74 kg ha^−1^), number of dry grains (13.00 to 16.55), 100-dry-grain weight (11.96 to 22.93 g), and dry grain yield (1059 to 3128.88 kg ha^−1^).

The GCA estimates for the 14 evaluated traits (Table 2) showed both positive and negative effects, with statistically significant estimates predominating across the traits analyzed. Among the evaluated landraces, L1, L3, and L5 exhibited the highest numbers of traits with significant GCA estimates. L1 had the highest GCA values for stem diameter and number of green grains, whereas L3 had the highest value for leaflet area. L5 excelled in 100-dry-grain weight, an economically important trait associated with commercial value.

SCA estimates varied among the evaluated traits, with both positive and negative effects observed (Table 3). The hybrid combination L4 × L5 showed the highest number of significant SCA effects, totaling nine traits, with excellent performance for stem diameter, leaflet area, plant height, central leaflet width, number of pods per plant, green pod length, green grain yield, 100-dry-grain weight, and dry grain yield. The hybrid L1 × L5 showed superior SCA effects for 100-green-grain weight, 100-dry-grain weight, dry pod length, and dry grain yield. Likewise, the hybrid combination L1 × L4 exhibited favorable effects for six traits, particularly central leaflet width, number of pods per plant, green grain yield, dry pod length, number of dry grains, and dry grain yield.

The reciprocal effect estimates (*rjj*) (Table S3—Supplementary Materials), showed both positive and negative values for the traits, stem diameter, plant height, central leaflet length and width, green pod length, and number and weight of dry grains. The results indicate variation in the direction of reciprocal effects. Statistically significant reciprocal effects were observed for seven traits, consistent with the effects identified in the analysis of variance.

The reciprocal cross L2 × L1 showed the highest number of significant effects, with nine traits, highlighting stem diameter, leaflet area; plant height; central leaflet length; central leaflet width; green pod length; number of green grains, 100-green-grain weight, green grain yield, dry pod length, 100- dry-grains weight, dry grain yield. Additionally, the reciprocal cross L3 × L2 exhibited significant effects for four traits, namely leaflet area, central leaflet length, number of pods per plant, number of green grains, 100-green-grain weight, green grain yield, dry pod length, number of dry grains, 100-dry-grains weight, dry grain yield.

The means for each trait of the hybrids, the reciprocals, and their respective parents are presented in Table S4 (Supplementary Materials).

**Figure 1:**
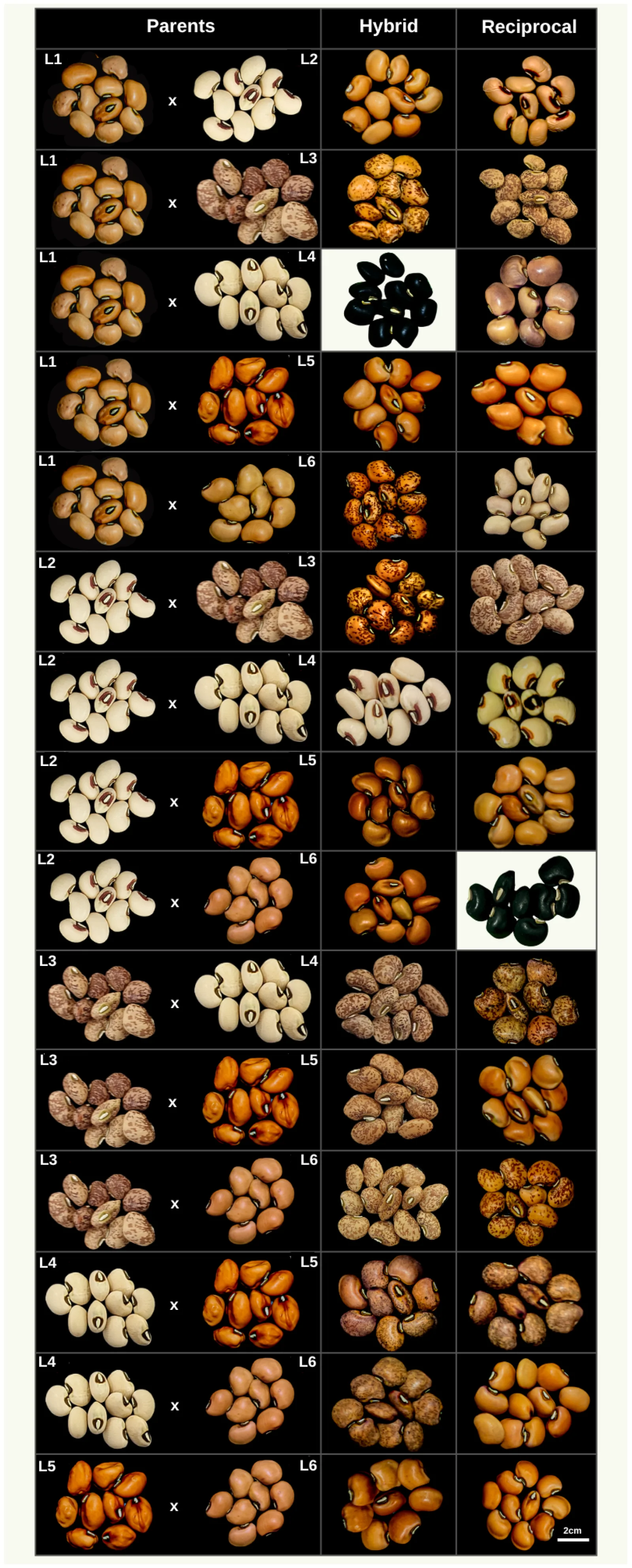
Seed coat color variation among 15 F_1_ hybrids and their 15 reciprocal crosses derived from six cowpea (*Vigna unguiculata*) landraces. In each pair, the genotype on the left was used as the female parent, whereas the genotype on the right served as the pollen donor. Hybrid combinations are identified together with their respective reciprocal crosses. Scale bar = 2 cm.

Variations in seed coat and hilum color were observed among the 15 hybrid combinations and their 15 reciprocal crosses (Table 4). Brown was the predominant color, occurring in 56.67% of both hybrids and reciprocal crosses, indicating broad commercial acceptance. New colors were also observed, including variegated (23.33%), beige (16.67%), black (6.67%), and white (6.67%), demonstrating morphological and agronomic diversity generated by the diallel crosses (Figure 1).

## 3. Discussion

The diallel analyses revealed significant effects of general combining ability (GCA), specific combining ability (SCA), and reciprocal effects, indicating that both parental selection and cross direction are critical for maximizing genetic gain in cowpea breeding [21,22]. These findings provide valuable guidance for parent selection and hybridization strategies, particularly for breeding programs targeting semiarid environments.

Although genomic and molecular approaches have expanded the tools available for modern crop breeding, diallel analysis remains useful when the objective is to quantify the combining ability of selected parents and identify superior cross combinations based on progeny performance. General combining ability (GCA), specific combining ability (SCA), and reciprocal effects provide complementary information on the contribution of parental and cross-specific effects to the expression of agronomic traits [23].

The simultaneous occurrence of additive, non-additive, and maternal effects highlights the complex genetic architecture underlying the evaluated traits. The predominance of GCA effects for grain yield, number of pods per plant, and plant height indicates that additive gene action plays a major role in the inheritance of these traits. In predominantly self-pollinating species such as cowpea, additive genetic effects favor breeding strategies based on selection in segregating generations, because they facilitate the fixation of favorable alleles and the development of superior lines with greater yield stability [24,25]. Therefore, breeding methods based on pedigree selection or single-seed descent (SSD) appear particularly suitable for exploiting the genetic variability identified here.

Positive correlations indicate that traits tend to vary in the same direction, whereas negative correlations reflect inverse relationships. These associations are relevant to plant breeding, as they may influence correlated responses to selection and help identify favorable relationships or potential antagonisms among the evaluated traits [26].

The predominance of general combining ability (GCA) effects for central valve width, green pod length, weight of 100 green seeds, and weight of 100 dry seeds indicate that the observed variation for these traits is largely associated with additive genetic effects. This pattern is relevant for plant breeding, as additive effects are more effectively exploited through selection and can be accumulated and fixed over generations. Thus, parents with high GCA can serve as sources of favorable alleles for the formation of segregating populations. In predominantly self-pollinating species, such as cowpea, successive selection in advanced generations favors the identification and fixation of superior allelic combinations. Therefore, the results indicate that, for these traits, the selection of parents with high combinatorial capacity may be more important for the development of superior lines than the isolated exploitation of non-additive effects [24,27].

Among the combinations evaluated, L1, L3, and L5 had the highest positive estimates of GCA for stem diameter, leaf area, number of green grains, and weight of 100 green grains. The results obtained highlight the importance of local varieties as sources of genetic variability for plant breeding and for the maintenance of genetic diversity on farms. The incorporation of these resources into breeding programs can be linked to farmers’ active participation in defining selection criteria, evaluating populations, and identifying traits of local importance. This approach allows for the integration of farmers’ knowledge with agronomic criteria, promoting the selection of materials adapted to growing conditions and community needs. In this way, participatory genetic improvement can contribute to the enhancement, continued use, and conservation of genetic diversity adapted to local conditions [28,29,30,31].

**Figure 2:**
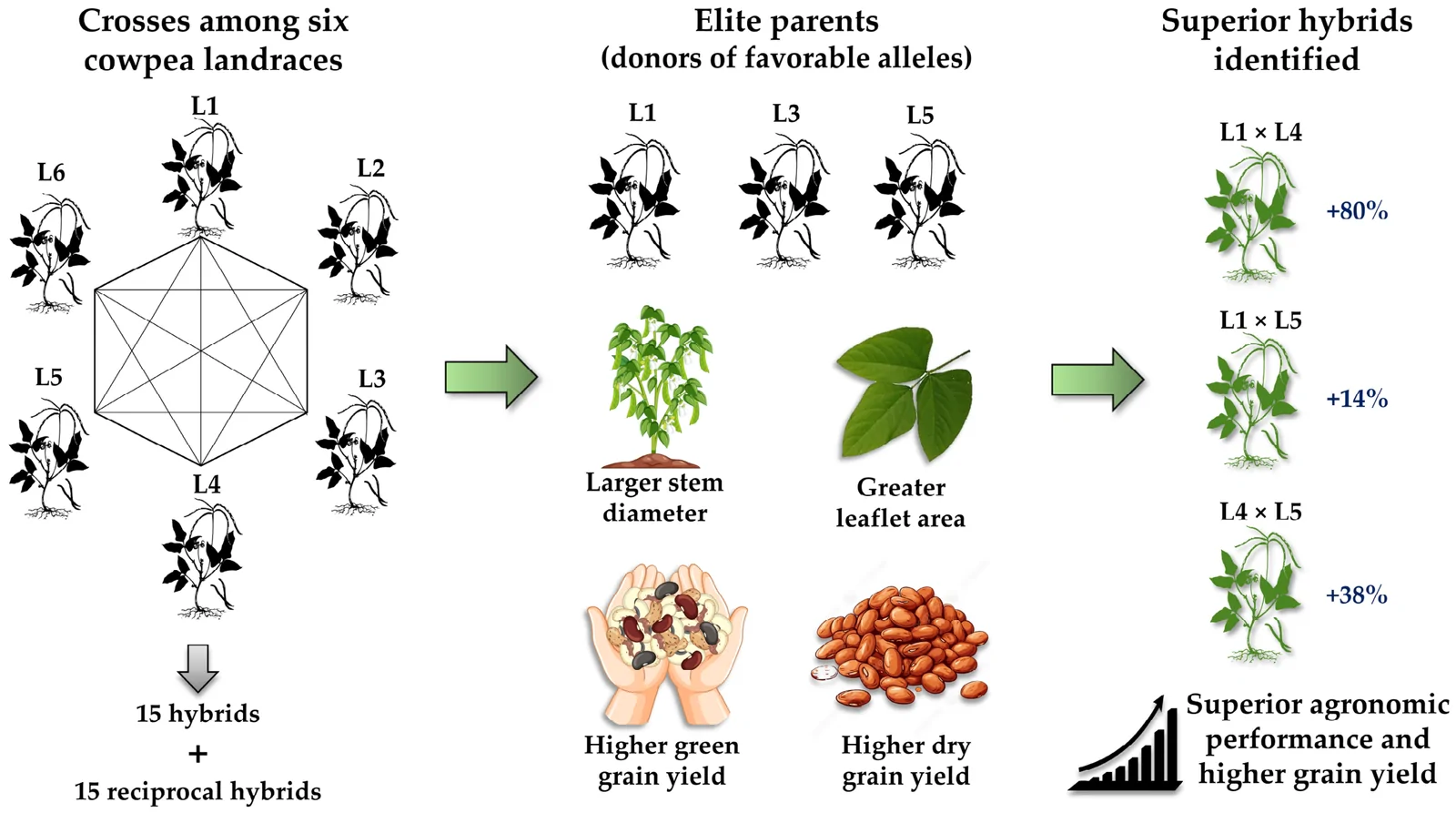
Diallel analysis identified superior parents and high-performing hybrid combinations among six cowpea (*Vigna unguiculata*) landraces, highlighting promising genetic resources for breeding under semiarid conditions. Percentage values represent hybrid grain yield relative to the mean of the parents. Percentage values were calculated as: [(hybrid grain yield − mean parental grain yield)/mean parental grain yield] × 100.

The hybrids L1 × L4, L1 × L5, and L4 × L5 combined significant positive SCA effects with superior phenotypic performance for grain yield and several yield-related traits, demonstrating strong agreement between combining ability estimates and field performance (Figure 2). The convergence of these two lines of evidence strengthens the confidence that these crosses exhibit high genetic complementarity and stand out for their superior performance in yield-related traits, indicating their potential as sources of genetic variability for plant breeding. for selecting superior recombinant lines in subsequent generations. Rather than indicating potential for commercial F_1_ hybrid cultivation, these results identify parental combinations with high potential to generate segregating populations enriched with desirable genotypes [29,31].

The percentage values shown in Figure 2 represent the deviation of hybrid grain yield from the mean performance of its two parents. Thus, positive values indicate that the hybrid performed above the parental mean. These values reflect relative hybrid performance and should not be interpreted as absolute yield gains or as evidence of superiority over the better-performing parent.

The superior agronomic performance of these combinations further supports their breeding value. Grain yields ranged from 1731.94 to 4640.74 kg ha^−1^, exceeding the reported for widely cultivated cowpea cultivars under semiarid conditions. For example, Rolim et al. [32] reported grain yields of approximately 403 kg ha^−1^ for the cultivar IPA 207, whereas Freire Filho et al. [33] and Costa et al. [34] indicated that cultivars such as IPA 207, BRS Pujante, and Canapu generally exceed 1000 kg ha^−1^ under irrigated conditions [35]. Although direct comparisons should be interpreted with caution because of differences in environmental conditions and crop management, these results demonstrate the considerable breeding potential of the evaluated hybrids combinations.

Significant reciprocal effects were detected for several traits, indicating that the direction of the cross may influence trait expression. These reciprocal effects may arise from different sources, including maternal, cytoplasmic, maternal environmental, or other parent-of-origin effects. Therefore, cross direction should be considered when designing hybridization schemes [24,36]. Similar findings have been reported in previous diallel studies in cowpea, reinforcing the importance of reciprocal effects when selecting parent combinations for breeding [3].

In addition to the variability identified for agronomic traits, substantial morphological diversity was detected in seed coat and hilum coloration among the hybrids and their reciprocal crosses. Besides increasing phenotypic variability, seed coat color is an economically important trait because it directly influences consumer preference and market segmentation across of Africa, Asia, and the Americas [37]. The appearance of new color patterns, including variegated, beige, black, and white seed coats, reflects the genetic recombination generated by the diallel crosses and expands the opportunities to select cultivars that simultaneously meet market demands while broadening the genetic diversity available to breeding programs.

The identification of superior parents and hybrid combinations derived from landraces is particularly relevant because cowpea breeding has historically received considerably less investment than major cereal crops. Consequently, cowpea breeding progress continues to depend heavily on the effective use of local genetic resources to broaden its breeding pool and improve adaptation to increasingly variable environmental conditions [38,39,40,41,42]. Our results demonstrate that farmer-conserved landraces remain an invaluable source of genetic diversity, contributing favorable alleles for grain yield, adaptation, and resilience under semiarid conditions [17].

Our findings demonstrate that cowpea landraces constitute strategic genetic resources for breeding programs aimed at improving grain yield and adaptation under semiarid conditions. The identification of parents with favorable traits and hybrid combinations with high genetic complementarity indicates their potential as parental sources in crosses designed to recombine traits of interest, particularly grain yield and its components. As an initial stage of a participatory breeding program, this study establishes the genetic basis for future selection cycles. This study represents an important first step in the participatory genetic improvement program for cowpea, as it identifies promising parental combinations that still require evaluation in subsequent generations to confirm their stability and identify superior genotypes.

Despite the results obtained in this study, it is important to acknowledge that the experiment was conducted at a single location and during a single growing season, which limits the extrapolation of the findings to other environmental conditions within the semiarid region. Therefore, the patterns of performance and stability observed should be interpreted within the context of the specific conditions evaluated. Further studies involving multiple locations and growing seasons are needed to confirm the consistency of these results and to provide a more comprehensive assessment of genotype × environment interactions, as well as the adaptation and stability of the genotypes under different semiarid conditions.

## 4. Materials and Methods

### 4.1. Selection of Genotypes

The cowpea landraces were selected using a participatory approach. Initially, 17 landraces were collected through surveys and prospecting in family farming communities. Farmers identified the preferred landraces based on traditional knowledge and local production systems. Six landraces were subsequently selected based on previous agronomic evaluations conducted within the Nexus Caatinga Project (www.nexuscaatinga.com.br/) (genotype × environment interaction and stability in landraces of cowpea under dryland conditions), as described by Rolim et al. [17], considering grain yield, biomass production, seed coat color, seed weight, adaptability, and yield stability under semiarid conditions (Table 5).

### 4.2. Hybridization and Evaluation of Hybrids

Before hybridization, two generations of the selected landraces were advanced through self-pollination and selection to increase genetic uniformity before diallel crossing. The six landraces were used as parents in a complete diallel design, generating 30 genotypes comprising 15 direct hybrids and 15 reciprocal crosses.

Hybridizations were performed using plants grown in the greenhouse of the Department of Biosciences, Center for Agricultural Sciences, Federal University of Paraíba (UFPB), Paraíba, Brazil (6°58′ S, 35°41′ W). The experimental site is located at 618 m above sea level under a humid tropical climate, with a rainy season in autumn and winter, an average annual temperature of 23 °C, relative humidity of around 80%, and mean annual rainfall of 1400 mm [45]. Staggered sowing was carried out to ensure a continuous supply of flowering plants throughout the crossing period. Standard crop management practices for cowpea were adopted. The chemical analysis of the soil before sowing revealed the following characteristics: pH in water (1:2.5) = 6.60; available phosphorus (P) and potassium (K^+^) = 73.97 and 119.95 mg dm^−3^, respectively; exchangeable calcium (Ca^2+^), magnesium (Mg^2+^), and sodium (Na^+^) = 8.99, 5.37, and 0.17 cmol dm^−3^; sum of exchangeable bases (SB = Ca^2+^ + Mg^2+^ + K^+^ + Na^+^) = 14.53 cmolc dm^−3^; potential acidity (H^+^ + Al^3+^) = 1.39 cmolc dm^−3^; cation exchange capacity (CEC = SB + H^+^ + Al^3+^) = 16.22 cmolcdm^−3^; and base saturation percentage = 91%.

Manual crosses were performed using flower buds at the balloon stage, characterized by a greenish-yellow color, flexible texture, and indehiscent anthers. Recipient buds were manually emasculated to prevent self-pollination. Pollen was collected from donor flowers at anthesis in the morning and stored under refrigeration until pollination. Crosses were made in the late afternoon, depending on bud availability, following adaptations of the techniques described by Freire Filho et al. [44] and Rêgo et al. [46]. After pollination, each bud was labeled to identify the respective cross. Plants were monitored throughout fruit development, and mature pods were harvested approximately 15 to 20 days after pollination, depending on the genotype.

**Figure 3:**
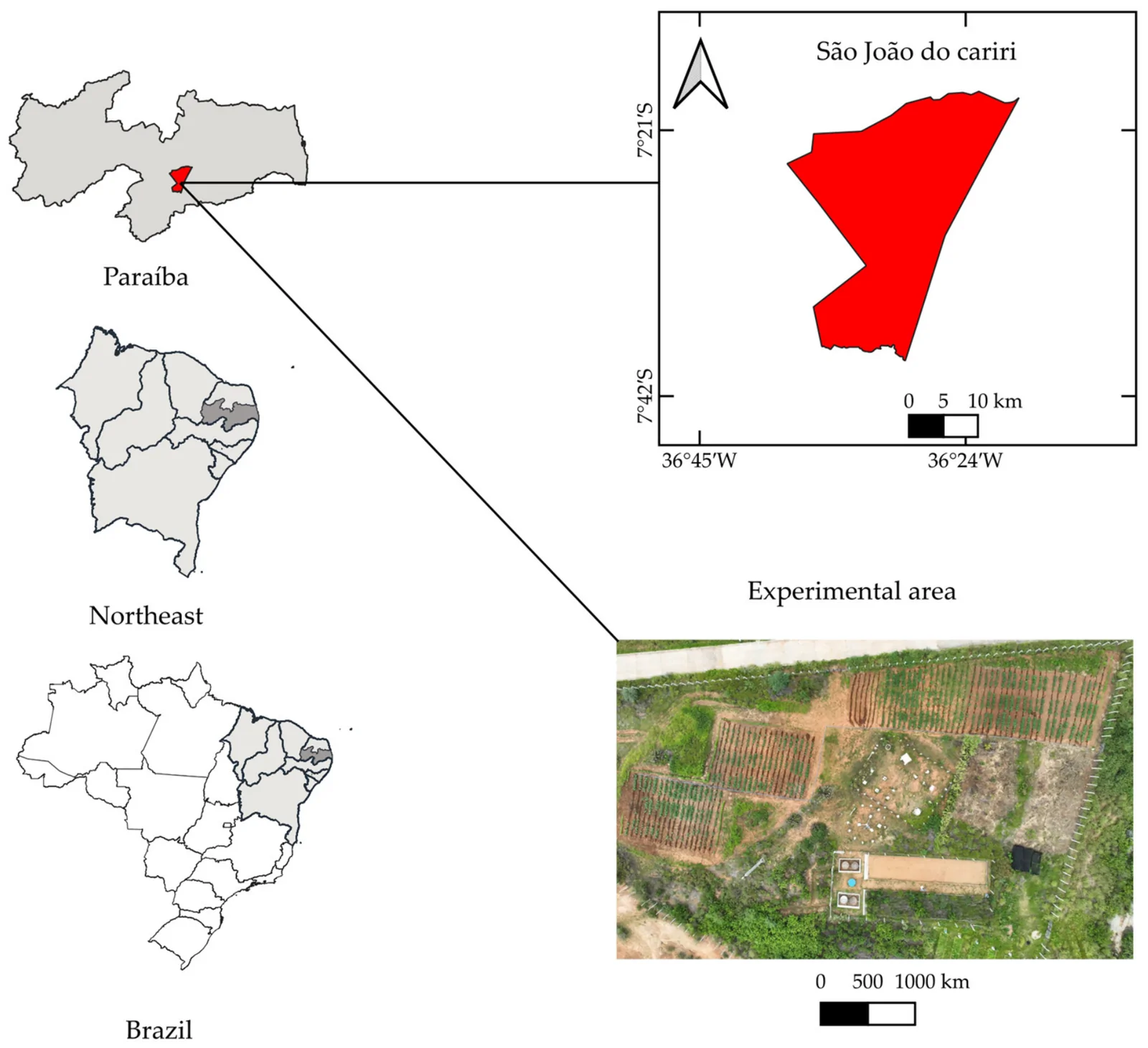
Location of the experimental field in the Cariri Paraibano region, Paraíba, Brazil, where six parents, 15 F_1_ hybrids, and 15 reciprocal crosses of cowpea (*Vigna unguiculata*) landraces were evaluated.

Seeds from the six parents and the F_1_ hybrid combinations including the respective reciprocal crosses, totaling 36 treatments were sown under rainfed conditions at the UFPB São João do Cariri Experimental Station in Paraíba, Brazil (7°23′ 30″ S, 36°31′59″ W) (Figure 3). The experimental area is located within the Brazilian semiarid region, one of the driest regions in the country, at an altitude ranging from 400 to 600 m above sea level. The climate is characterized by a mean annual temperature of 25 °C, annual rainfall around 400 mm [47], and relative humidity of about 70%. The soils are shallow, moderately to sandy texture, with a gravelly surface, and classified as Luvisols.

During the field experiment, conducted from March to July 2023, cumulative rainfall reached 401 mm, corresponding to approximately 77.2% of the annual precipitation (519.4 mm). During this period, the mean air temperature, relative humidity, and soil temperature were 24.4 °C, 75%, and 28.6 °C, respectively (Table S5—Supplementary Materials). Meteorological data were obtained from the weather station located at the experimental site.

The soil was conventionally prepared using a disk harrow two weeks before sowing. Seeds were sown manually, with one seed per hole. Topdressing fertilization was applied 32 days after sowing, using 30 kg ha^−1^ of nitrogen (N source) and 40 kg ha^−1^ of potassium (K source). Weeds were controlled manually by hoeing. Insect pests were managed using natural extracts such as mineral oil, neem oil, a 1% neutral detergent solution, and *Beauveria bassiana* (Boveril) applied at 15 g per 10 L of water. Granular ant bait was applied manually at a rate of 10 g m^−2^.

Each experimental plot consisted of three rows, each 4.0-m-long, spaced 1.0 m apart, with 0.40 m between plants, totaling approximately 40 plants per plot. The central row, comprising approximately 10 plants, was considered the experimental unit, and all plants within this row were evaluated. The evaluations were conducted according to the developmental stage of each genotype, taking into account the formation of immature pods prior to physiological maturity. The early-maturing varieties were harvested at 60 days, while the intermediate and late-maturing varieties were harvested between 65 and 75 days. At physiological maturity, harvesting took place at 80 days for early and intermediate genotypes and between 85 and 90 days for late genotypes. Pod and seed descriptors were evaluated only in the plants from the central row.

The evaluated descriptors followed the minimum requirements established for determining the *Value for Cultivation and Use (VCU)* of *V. unguiculata* according to the *National Register of Cultivars (RNC)* [47]. Quantitative traits included stem diameter (cm), leaflet area (cm^2^), plant height (cm), central leaflet length (cm), central leaflet width (cm), number of pods per plant, green pod length (cm), number of grains per green pod, 100-green-grain weight (g), green grain yield (kg ha^−1^), dry pod length (cm), number of grains per dry pod, 100-dry-grain weight (g), and dry grain yield (kg ha^−1^). Leaflet area was determined using ImageJ^®^ software (version 1.54p) during the fruiting stage.

### 4.3. Experimental Design and Statistical Analysis

The experimental followed a randomized complete block design with ten replications. Data were initially tested for normality and subsequently subjected to analysis of variance (ANOVA). Treatment means were grouped using the Scott–Knott test at a 5% significance level. Combining ability analysis was performed according to Griffing’s, Method I (fixed-effects model) [48], allowing estimation of general combining ability (GCA) and specific combining ability (SCA).

Mean square effects between parents and hybrids (s_ij_) were compared using the *t*-test. All statistical analyses were performed using GENES software (version 1990.2023.15) [48].

Seed coat color was quantified visually for each color category. The relative frequency (%) of each color was calculated as:

$$\mathrm{Relative}\;\mathrm{frequency}\;(\%)=\frac{\mathrm{Number}\;\mathrm{of}\;\mathrm{seeds}\;\mathrm{of}\;a\;\mathrm{given}\;\mathrm{color}}{\mathrm{Total}\;\mathrm{number}\;\mathrm{of}\;\mathrm{seeds}\;\mathrm{evaluated}}\times 100$$

## 5. Conclusions

Cowpea landraces of *V. unguiculata* exhibited substantial genetic variability, confirming their value as genetic resources for breeding programs. The parental landraces L1, L3, and L5 were identified as superior donors of favorable alleles for agronomic traits, whereas the hybrid combinations L1 × L4, L1 × L5, and L4 × L5 showed superior performance for plant growth and grain yield. These findings demonstrate the usefulness of diallel analysis for identifying superior parents and promising hybrid combinations, and provide valuable genetic resources for developing cowpea cultivars adapted to semiarid conditions.

## Figures and Tables

**Table 1:** Analysis of variance for 14 yield-related traits evaluated from a diallel cross among six parents and their F_1_ hybrids of cowpea (*Vigna unguiculata*) landraces evaluated under semiarid conditions in northeastern Brazil.

**Source**	**DF**	**SD**	**LA**	**PH**	**CLL**	**CLW**	**NPP**	**GPL**
Treatment	35	0.2 **	5188.3 **	6714.3 **	6.3 **	3.1 **	225.1 **	25.6 **
GCA	5	0.8 **	15,196.9 **	17,394.4 **	23.0 **	8.2 **	57.7 ^ns^	117.7 **
SCA	15	0.2 **	5109.3 **	5849.8 **	3.7 **	2.9 **	382.6 **	9.6 **
Reciprocal	15	0.1 **	1931.1 ^ns^	4018.7 **	3.41 **	1.7 *	123.4 ^ns^	10.8 **
Residue	280	0.0	1563.2	1896.0	1.41	0.8	112.8	4.5
*σ* ^2^ *_G_*		0.0	126.2	143.5	0.20	0.6	−0.50	1.0
*σ* ^2^ *_s_*		0.1	394.0	439.3	0.24	0.0	29.9	0.5
*σ* ^2^ *_E_*		0.0	20.4	117.9	1.47	0.8	0.59	0.3
*CV_g_*/*CV_e_*		0.0	0.3	0.3	0.8	14.7	−0.01	2.0
CV		19.0	18.7	54.4	9.94	11.1	46.2	9.96
**Source**	**DF**	**NGG**	**100-GGW**	**GGY**	**DPL**	**NDG**	**100-DGW**	**DGY**
Treatment	35	9.7 **	101.5 **	5,598,330.4 **	16.5 **	9.1 **	25.3 **	2,212,175.0 **
GCA	5	23.4 **	485.0 **	12,095,587.2 **	43.8 **	14.2 *	98.5 **	2,176,936.7 ^ns^
SCA	15	9.6 *	50.2 *	6,350,829.6 **	17.9 **	7.2 ^ns^	9.9 ^ns^	2,640,970.2 **
Reciprocal	15	5.2 ^ns^	25.0 ^ns^	2,680,078.9 ^ns^	6.0 ^ns^	9.3 *	16.2 *	1,795,125.8 ^ns^
Residue	280	5.3	25.2	2,853,079.5	5.2	5.2	7.7	1,089,628.7
*σ* ^2^ *_G_*		0.1	4.2	85,578.7	0.35	0.0	0.8	10,067.6
*σ* ^2^ *_s_*		0.4	2.7	388,638.8	1.4	0.2	0.2	172,371.2
*σ* ^2^ *_E_*		0.0	0.0	9611.1	0.0	0.2	0.4	1,089,628.7
*CV_g_*/*CV_e_*		0.3	1.56	0.2	0.0	0.3	3.4	0.0
CV		15.9	29.8	52.9	10.8	15.6	26.4	51.7

* and ** = significant at 5% and 1% probability levels, respectively, by the F-test. ^ns^: not significant; SV: source of variation; GCA: general combining ability; SCA: Specific combining ability; *σ*^2^*_G_* = genetic variance; *σ*^2^*_s_* = environmental variance; *σ*^2^*_E_* = genetic standard deviation; *CV_g_*/*CV_e_* = genetic variance/environmental variance; CV: coefficient of variation; DF: degree of freedom, SD: stem diameter; LA: leaflet area; PH: plant height; CLL: central leaflet length; CLW: central leaflet width; NPP: number of pods per plant; GPL: green pod length; NGG: number of green grains, 100-GGW: 100-green-grain weight; GGY: green grain yield; DPL: dry pod length; NDG: number of dry grains; 100-DGW: 100-dry-grain weight; DGY: dry grain yield.

**Table 2:** Estimates of general combining ability (GCA) for 14 agronomic traits of six parents used in diallel crosses of cowpea (*Vigna unguiculata*) landraces evaluated under semiarid conditions in northeastern Brazil.

**Parents**	**GCA**						
	**SD**	**LA**	**PH**	**CLL**	**CLW**	**NPP**	**GPL**
L1	0.11 **	5.32 **	6.26 **	−0.01 ^ns^	0.31 **	0.17 ^ns^	−0.39 **
L2	−0.09 **	−9.91 **	−7.66 **	−0.43 **	−0.16 **	−0.85 **	−0.78 **
L3	−0.03 **	21.04 **	−3.49 **	0.74 **	0.33 **	−0.35 ^ns^	1.80 **
L4	−0.03 **	−0.82 ^ns^	−19.32 **	0.34 **	−0.26 **	1.23 **	−1.04 **
L5	−0.11 **	−5.43 **	8.33 **	0.29 **	0.44 **	−0.44 ^ns^	0.54 **
L6	0.04 **	−10.19 **	15.88 **	−0.34 **	−0.26 **	0.24 ^ns^	−0.12 **
**Parents**	**GCA**						
	**NGG**	**100-GGW**	**GGY**	**DPL**	**NDG**	**100-DGW**	**DGY**
L1	0.33 **	1.94 **	356.37 **	−0.34 **	0.16 **	0.63 **	61.71 *
L2	−0.40 **	−2.23 **	453.29 **	−0.43 **	−0.25 **	−0.94 **	207.64 **
L3	0.03 ^ns^	1.82 **	295.74 **	1.15 **	−0.51 **	0.55 **	107.20 **
L4	−0.64 **	−2.91 **	351.98 **	−0.43 **	−0.12 *	−1.14 **	86.80 **
L5	0.62 **	1.35 **	123.52 **	0.34 **	0.45 **	1.23 **	178.85 **
L6	0.05 ^ns^	0.02 ^ns^	29.64 ^ns^	−0.28 **	0.28 **	−0.34 **	53.33 **

* and ** Significant at the 5% and 1% probability levels, respectively, by *t*-test. ^ns^: not significant, SD: stem diameter; LA: leaflet area; PH: plant height; CLL: central leaflet length; CLW: central leaflet width; NPP: number of pods per plant; GPL: green pod length; NGG: number of green grains, 100-GGW: 100-green-grain weight; GGY: green grain yield; DPL: dry pod length; NDG: number of dry grains; 100-DGW: 100-dry-grains weight; DGY: dry grain yield.

**Table 3:** Estimates of specific combining ability (SCA) for 14 agronomic traits in 15 hybrid combinations from diallel crosses among six cowpea (*Vigna unguiculata*) landraces evaluated under semiarid conditions in northeastern Brazil.

Hybrids	SCA													
	SD	LA	PH	CLL	CLW	NPP	GPL	NGG	100-GGW	GGY	DPL	NDG	100-DGW	DGY
L1 × L2	0.05 **	11.57 **	−2.73 ^ns^	0.20 **	0.26 **	0.35 ^ns^	0.59 **	1.05 **	1.57 **	543.41 **	0.45 **	0.52 **	0.35 *	191.52 **
L1 × L3	−0.05 **	−18.13 **	−32.23 **	−0.16 *	0.26 **	−0.64 ^ns^	−1.10 **	−1.07 **	−1.78 **	−354.60 **	0.13 ^ns^	−0.27 *	0.94 **	244.91 **
L1 × L4	0.02 ^ns^	−29.70 **	1.14 ^ns^	−0.60 **	0.14 **	9.09 **	−0.93 **	−0.36 **	−2.94 **	310.66 **	0.29 *	0.28 *	0.29 ^ns^	640.12 **
L1 × L5	−0.08 **	−1.61 ^ns^	3.15 ^ns^	−0.60 **	−0.71 **	−3.33 **	−0.09 ^ns^	−0.36 **	2.24 **	−46.09 ^ns^	0.64 **	−0.46 **	1.08 **	424.78 **
L1 × L6	0.09 **	19.00 **	22.49 **	0.40 **	−0.41 **	4.53 **	−0.30 **	−0.16 ^ns^	−2.94 **	872.59 **	0.20 **	−0.67 **	1.20 **	36.98 **
L2 × L3	−0.01 ^ns^	1.89 ^ns^	−11.75 **	−0.05 ^ns^	0.39 **	−0.38 ^ns^	−0.08 ^ns^	−0.16 ^ns^	−0.29 ^ns^	−251.83 **	−2.13 ^ns^	−0.18 ^ns^	0.47 **	−126.51 *
L2 × L4	−0.06 **	−0.50 ^ns^	0.67 ^ns^	0.16 *	0.24 **	−2.08 **	−0.67 **	−0.29 *	−1.25 **	150.66 **	0.56 **	0.58 **	0.51 **	−86.52 ^ns^
L2 × L5	−0.01 ^ns^	19.68 **	−13.31 **	0.86 **	0.56 **	2.70 **	0.08 ^ns^	0.10 ^ns^	1.89 **	−152.38 **	0.45 **	−0.32 *	0.07 ^ns^	144.89 *
L2 × L6	−0.10 **	−10.42 **	−4.43 ^ns^	−0.34 **	−0.75 **	−1.65 **	−0.12 ^ns^	0.59 **	−2.01 **	−251.29 **	1.03 **	−0.48 **	0.65 **	−222.12 **
L3 × L4	0.27 **	0.00 ^ns^	20.18 **	−0.29 **	−0.07 ^ns^	−0.80 ^ns^	0.14 ^ns^	−0.05 ^ns^	−0.33 ^ns^	−113.42 ^ns^	0.08 ^ns^	−0.70 **	0.21 ^ns^	−202.62 **
L3 × L5	0.04 **	−3.19 ^ns^	9.85 **	0.24 **	0.02 ^ns^	1.36 *	−0.08 ^ns^	−0.06 ^ns^	−0.68 *	42.82 **	0.37 **	−0.29 *	0.74 **	40.04 **
L3 × L6	−0.09 **	10.22 **	9.53 **	0.13 *	−0.12 *	0.84 ^ns^	1.68 **	1.41 **	−0.67 *	425.08 **	1.57 **	1.27 **	0.39 *	175.01 **
L4 × L5	0.19 **	18.44 **	20.23 **	−0.03 ^ns^	0.24 **	6.38 **	0.23 *	−0.26 *	0.42 ^ns^	1064.03 **	0.05 ^ns^	0.09 ^ns^	0.30 *	667.15 **
L4 × L6	−0.09 **	5.36 ^ns^	−25.80 **	0.00 ^ns^	0.21 **	−4.57 **	−0.41 **	−0.14 ^ns^	−0.00 ^ns^	−658.34 **	0.58 **	0.66 **	0.72 **	−233.24 **
L5 × L6	0.10 **	3.56 ^ns^	10.17 **	−0.08 ^ns^	0.19 **	−4.79 **	0.35 **	−1.72 **	−1.15 **	−873.85 **	0.71 **	0.30 *	0.62 **	−338.06 **

* and ** significant at the 5% and 1% probability levels, respectively, by the *t*-test. ^ns^: not significant; SD: stem diameter; LA: leaflet area; PH: plant height; CLL: central leaflet length; CLW: central leaflet width; NPP: number of pods per plant; GPL: green pod length; NGG: number of green grains, 100-GGW: 100-green-grain weight; GGY: green grain yield; DPL: dry pod length; NDG: number of dry grains; 100-dry-grains weight; DGY: dry grain yield.

**Table 4:** Morphological and agronomic characterization of 15 F_1_ hybrids and their 15 reciprocal crosses of cowpea (*Vigna unguiculata*) landraces, evaluated under semiarid conditions in northeastern Brazil.

Hybrids	Traits		Reciprocal	Traits	
	SCC	HC		SCC	HC
L1 × L2	Brown	Light brown	L2 × L1	Beige	Brown
L1 × L3	Variegated	Light brown	L3 × L1	Variegated	Brown
L1 × L4	Black	Black	L3 × L2	Variegated	Brown
L1 × L5	Beige	Brown	L4 × L1	Beige	Brown
L1 × L6	Variegated	Brown	L4 × L2	White	Black
L2 × L3	Brown	Light brown	L4 × L3	Variegated	Brown
L2 × L4	Variegated	Light brown	L5 × L1	Beige	Brown
L2 × L5	Brown	Black	L5 × L2	Beige	Brown
L2 × L6	Beige	Brown	L5 × L3	Variegated	White
L3 × L4	Variegated	Brown	L5 × L4	Variegated	Brown
L3 × L5	Variegated	Brown	L6 × L1	Brown	Brown
L3 × L6	Variegated	Brown	L6 × L2	Black	Brown
L4 × L5	Variegated	Brown	L6 × L3	Variegated	White
L4 × L6	Variegated	Brown	L6 × L4	Beige	Brown
L5 × L6	Brown	Brown	L6 × L5	Beige	White

SCC: seed coat color; HC: hilum color.

**Table 5:** Description of six cowpea (*Vigna unguiculata*) landraces from the Brazilian semiarid region, selected for adaptation, grain yield, and earliness.

Genotypes	Collection Location	Genetic Base	Class ^1^	Subclass ^2^
L1	Aurora/CE	Landrace	Colors	Mulato Liso
L2	Remígio/PB	Landrace	White	Fradinho
L3	Prata/PB	Landrace	Colors	Corujinha
L4	Alagoa Grande/PB	Landrace	White	Fradinho
L5	Pocinhos/PB	Landrace	Colors	Mulato Liso
L6	Prata/PB	Landrace	Colors	Mulato Liso

CE: State of Ceará, Brazil; PB: State of Paraíba, Brazil; ^1^ Commercial grain-color class, Normative Instruction No. 12, dated 03/28/2008 [43]; ^2^ Commercial subclass based on grain tegument traits by Freire Filho et al. [44].

## Data Availability

The original contributions presented in this study are included in the article/Supplementary Materials. Further inquiries can be directed to the corresponding author.

## Supplementary Materials

The following supporting information can be downloaded at: https://www.mdpi.com/article/10.3390/plants15203145/s1, Table S1: Mean simple linear correlation coefficients for the 14 yield-related traits, evaluated from a diallel cross between six parents and their F_1_ hybrids of cowpea landraces (*Vigna unguiculata*), evaluated under semiarid conditions in northeastern Brazil. Table S2: Variation of 14 traits evaluated between cowpea (*Vigna unguiculata*) parents and hybrids grown in a semiarid region, Northeastern Brazil. Table S3: Estimate of the reciprocal effect (*rjj*), evaluated for 14 traits involving 15 possible combinations in diallel crosses among six cowpea (*Vigna unguiculata*) landraces cultivated in a semiarid region of Northeastern Brazil. Table S4: Comparison of 14 evaluated traits between parent lines and hybrids of cowpea (*Vigna unguiculata*) grown in a semiarid region of Northeast Brazil. Table S5: Climatic data recorded at the Experimental Farm of the Federal University of Paraíba (UFPB), located in the municipality of São João do Cariri, Paraíba, Brazil, during 2023.
