# Supplementary material for: Combining Ability and Hybrid Performance of Cowpea Landraces Under Semiarid Conditions
Source: Plants (Basel). 2026 Oct 14;15(20):3145. doi: 10.3390/plants15203145 (PMC13708615; doi:10.3390/plants15203145)
Supplement: Supplementary file 1 [file plants-15-03145-s001.zip › Table S1 - Supplementary Materials.pdf]

Supplementary Materials

Table S1: Mean simple linear correlation coefficients for the 14 yield-related traits, evaluated from a diallel cross between six parents and their F_1 hybrids of cowpea landraces (*Vigna unguiculata*), evaluated under semiarid conditions in northeastern Brazil.

**Traits**	**SD**	**LA**	**PH**	**CLL**	**CLW**	**NPP**	**GPL**	**NGG**	**100-GGW**	**GGY**	**DPL**	**NDG**	**100-DGW**	**DGY**
**SD**	1													
**LA**	0.201^{ns}	1												
**PH**	0.594**	0.053^{ns}	1											
**CLL**	-0.196^{ns}	0.725**	-0.284^{ns}	1										
**CLW**	0.325*	0.710**	0.038^{ns}	0.616**	1									
**NPP**	0.334*	-0.103^{ns}	0.066^{ns}	-0.148^{ns}	-0.155^{ns}	1								
**GPL**	0.085^{ns}	0.532**	0.202^{ns}	0.390*	0.345*	0.188^{ns}	1							
**NGG**	0.171^{ns}	0.229^{ns}	0.312^{ns}	0.053^{ns}	0.345^{ns}	-0.155^{ns}	0.773**	1						
**100-GGW**	0.380*	0.434**	0.390*	0.182^{ns}	0.184**	-0.256^{ns}	0.575**	0.179^{ns}	1					
**GGY**	0.575**	0.302^{ns}	0.358*	0.031^{ns}	0.477**	-0.229^{ns}	0.690**	0.687**	0.033^{ns}	1				
**DPL**	0.113^{ns}	0.434**	0.106^{ns}	0.405*	0.254^{ns}	0.630**	0.383*	0.282^{ns}	0.568**	0.192^{ns}	1			
**NDG**	0.267^{ns}	-0.039^{ns}	0.064^{ns}	-0.133^{ns}	0.346*	-0.022^{ns}	0.702**	0.260^{ns}	0.414*	0.280^{ns}	0.745**	1		
**100-DGW**	0.427**	0.220^{ns}	0.094^{ns}	0.167^{ns}	-0.048^{ns}	-0.052^{ns}	0.094^{ns}	0.399*	0.190^{ns}	0.079^{ns}	0.291^{ns}	0.323^{ns}	1	
**DGY**	0.511 **	0.048^{ns}	0.032^{ns}	-0.026^{ns}	0.536**	-0.126^{ns}	0.546**	0.476**	0.701**	0.386*	0.563**	0.405*	0.164^{ns}	1

^{ns} = not significant; * = significant at the 5% probability level; ** = significant at the 1% probability level. SD: Stem diameter; LA: Leaflet area; PH: Plant height; CLL: Central leaflet length; CLW: Central leaflet width; NPP: Number of pods per plant; GPL: Green pod length; NGG: Number of green grains, 100-GGW: 100-green-grain weight; GGY: green grain yield; DPL: dry pod length; NDG: Number of dry grains; 100-DGW: 100-dry-grain weight; DGY: dry grain yield.
