# Supplementary material for: Combining Ability and Hybrid Performance of Cowpea Landraces Under Semiarid Conditions
Source: Plants (Basel). 2026 Oct 14;15(20):3145. doi: 10.3390/plants15203145 (PMC13708615; doi:10.3390/plants15203145)
Supplement: Supplementary file 1 [file plants-15-03145-s001.zip › Table S2 - Supplementary Materials.pdf]

Supplementary Materials

Table S2. Variation of 14 traits evaluated between cowpea (*Vigna unguiculata*) parents and hybrids grown in a semiarid region, Northeastern Brazil.

Traits	Variation	Parents	Hybrids	Reciprocal
**SD**	0.98 – 1.61	L1, L3 and L6	L1 x L2, L1 x L3, L1 x L5, L1 x L6	L4 x L1, L4 x L5, L5 x L1, L5 x L4, L5 x L3, L5 x L6, L6 x L1, L6 x L3, L6 x L5
**LA**	162.68 – 298.28	L1 and L3	L1 x L2, L1 x L3, L1 x L4, L1 x L5, L1 x L6, L2 x L3, L2 x L4, L3 x L4, L3 x L5, L3 x L6, L4 x L5, L5 x L6	L2 x L1, L3 x L1, L3 x L2, L4 x L1, L4 x L3, L5 x L1, L5 x L2, L5 x L3, L5 x L4, L6 x L1, L6 x L3, L6 x L4
**PH**	24.88 – 143.33	L1, L2 and L6	L1 x L2, L1 x L3, L3 x L4, L3 x L5, L3 x L6, L4 x L5, L5 x L6	L4 x L1, L5 x L1, L5 x L2, L5 x L3, L6 x L1, L6 x L2, L6 x L3, L6 x L5
**CLL**	11.20 – 13.83	L1, L3 and L4	L1 x L2, L1 x L3, L2 x L3, L3 x L4, L3 x L5, L3 x L6, L4 x L5, L4 x L6	L3 x L1, L3 x L2, L4 x L1, L4 x L2, L4 x L3, L5 x L2, L6 x L1, L6 x L3
**CLW**	7.07 – 9.85	L1 and L3	L1 x L2, L1 x L3, L1 x L6, L2 x L3, L2 x L5, L3 x L5, L3 x L6, L4 x L5, L5 x L6	L3 x L1, L3 x L2, L5 x L1, L5 x L2, L5 x L3, L6 x L1
**NPP**	13.33 – 34.77	L6	L1 x L4, L1 x L6, L2 x L3, L2 x L5, L3 x L6, L4 x L5	L3 x L2, L4 xL3, L5 x L3, L5 x L4, L6 x L1
**GPL**	19.26 – 25.44	L3 and L5	L3 x L4, L3 x L5, L3 x L6, L5 x L6,	L6 x L3
**NGG**	10.40 – 16.33	L1 and L3	L1 x L2, L1 x L4, L1 x L5, L1 x L6, L2 x L5, L3 x L4, L3 x L5, L3 x L6, L4 x L6	L2 x L1, L5 x L1, L5 x L3, L5 x L6, L6 x L1, L6 x L2, L6 x L3, L6 x L5
**100-GGW**	11.96 – 22.93	L1 and L5	L1 x L2, L1 x L3, L1 x L5, L1 x L6, L3 x L5, L3 x L6	L3 x L2, L5 x L3, L6 x L1, L6 x L3
**GGY**	1731.94 – 4640.74	L3, L5 and L6	L1 x L2, L1 x L3, L1 x L4, L1 x L5, L1 x L6, L2 x L3, L3 x L5, L3 x L6, L4 x L5	L2 x L1, L3 x L1, L3 x L2, L4 x L1, L4 x L2, L5 x L1, L5 x L3, L5 x L4, L6 x L1, L6 x L2, L6 x L3
**DPL**	18.64 – 23.95	L2, L3, L4 and L5	L1 x L2, L1 x L3, L3 x L4, L3 x L5, L3 x L6, L4 x L2, L4 x L6, L6 x L2	L3 x L1, L4 x L3, L6 x L2
**NDG**	13.00 – 16.55	L1 and L5	L1 x L2, L1 x L4, L2 x L3, L3 x L5, L3 x L6, L4 x L6, L5 x L6	L2 x L1, L4 x L1, L5 x L1, L5 x L2, L5 x L3, L6 x L1, L6 x L3, L6 x L4, L6 x L5
**100-DGW**	7.18 – 13.96	L1, L3 and L5	L1 x L2, L1 x L3, L3 x L4, L3 x L5, L4 x L5, L5 x L6	L3 x L1, L3 x L2, L5 x L1, L5 x L2, L5 x L3, L5 x L4, L6 x L3,
**DGY**	1059.53 – 3128.88	L1, L3, L5 and L6	L1 x L2, L1 x L3, L1 x L4, L2 x L5, L2 x L6, L3 x L5, L3 x L6	L3 x L1, L3 x L2, L4 x L1, L5 x L1, L5 x L2, L5 x L3, L5 x L4, L6 x L2, L6 x L3

SD: Stem diameter; LA: Leaflet area; PH: Plant height; CLL: Central leaflet length; CLW: Central leaflet width; NPP: Number of pods per plant; GPL: Green pod length; NGG: Number of green grains, 100-GGW: 100-green-grain weight; GGY: green grain yield; DPL: dry pod length; NDG: Number of dry grains; 100-DGW: 100-dry-grains weight; DGY: dry grain yield.
