# Supplementary material for: Combining Ability and Hybrid Performance of Cowpea Landraces Under Semiarid Conditions
Source: Plants (Basel). 2026 Oct 14;15(20):3145. doi: 10.3390/plants15203145 (PMC13708615; doi:10.3390/plants15203145)
Supplement: Supplementary file 1 [file plants-15-03145-s001.zip › Table S3 - Supplementary Materials.pdf]

Table S3. Estimate of the reciprocal effect (*rjj*), evaluated for 14 traits involving 15 possible combinations in diallel crosses among six cowpea (*Vigna unguiculata*) landraces cultivated in a semiarid region of Northeastern Brazil.

Reciprocal	*rjj*													
	**SD**	**LA**	**PH**	**CLL**	**CLW**	**NPP**	**GPL**	**NGG**	**100-GGW**	**GGY**	**DPL**	**NDG**	**100-DGW**	**DGY**
L2 x L1	0.12**	7.50**	–21.16**	0.39**	0.48**	0.88^{ns}	0.78**	0.15**	1.75**	395.55**	0.50**	0.05^{ns}	2.20**	543.51**
L3 x L1	0.11**	12.07**	6.05^{ns}	0.06^{ns}	0.65**	0.94^{ns}	–0.38**	–0.46**	1.16**	230.83*	–0.32*	–0.33*	0.71**	286.01**
L3 x L2	–0.00^{ns}	5.11*	3.05^{ns}	–0.48**	–0.06^{ns}	–3.61**	0.24^{ns}	–0.2**	–0.75*	–580.04**	–0.32*	0.66**	1.21**	533.47**
L4 x L1	–0.00^{ns}	–5.60*	–14.05**	–0.48**	–0.33**	1.27**	0.27^{ns}	0.77**	0.51^{ns}	377.36**	0.15^{ns}	0.16^{ns}	0.56**	370.37**
L4 x L2	0.08**	8.71**	–4.66^{ns}	–0.54**	–0.25**	4.83**	0.24^{ns}	0.33^{ns}	0.42^{ns}	762.22**	–0.15^{ns}	0.16^{ns}	0.15^{ns}	337.86**
L4 x L3	–0.08**	–12.07**	3.11^{ns}	–0.17*	0.03^{ns}	–2.72**	1.21**	0.61^{ns}	0.51^{ns}	–218.56**	–0.04^{ns}	–0.72**	0.56**	–97.26^{ns}
L5 x L1	–0.01^{ns}	1.80^{ns}	15.50**	–0.47**	–0.23**	–1.05**	0.84**	0.11*	0.42^{ns}	–216.11^{ns}	1.33**	–1.77**	1.86**	305.55**
L5 x L2	–0.01^{ns}	–15.23**	–16.88**	–0.35**	–0.30**	3.05**	–0.76**	0.31^{ns}	0.92**	–223.84*	1.07**	–0.27^{ns}	1.20**	70.14**
L5 x L3	–0.16**	5.26*	–6.22^{ns}	0.82**	0.25**	–3.55**	1.46**	0.42**	–0.55^{ns}	–227.17*	0.14^{ns}	–0.83**	0.25^{ns}	401.16**
L5 x L4	–0.02*	13.86**	13.22**	0.77**	0.35**	–2.72**	0.28*	1.19^{ns}	2.21**	–489.90**	0.60**	0.72**	0.25^{ns}	351.67**
L6 x L1	–0.10**	–14.91**	14.38**	–0.45**	–0.32**	–0.61^{ns}	0.31*	0.12^{ns}	0.63^{ns}	192.59^{ns}	–0.28^{ns}	–0.72**	0.03^{ns}	297.17**
L6 x L2	–0.09**	–5.11*	–27.53**	0.18*	–0.15*	–3.16**	–0.59**	–0.84**	1.83**	–611.80**	0.23^{ns}	0.05^{ns}	0.24^{ns}	292.87**
L6 x L3	–0.18**	–14.86**	–4.22^{ns}	0.16*	0.21**	0.61^{ns}	–0.59**	0.00*	–0.49^{ns}	–247.96*	–0.40*	–0.88**	0.18^{ns}	65.04^{ns}
L6 x L4	–0.05**	–8.52**	–8.05^{ns}	0.26**	–0.08^{ns}	–0.77^{ns}	–0.05^{ns}	0.28^{ns}	–0.16^{ns}	–6.99^{ns}	0.07^{ns}	0.55**	0.27^{ns}	–64.55^{ns}
L6 x L5	–0.05**	11.40**	–28.96**	0.29**	0.27**	–0.11^{ns}	0.98**	0.67**	1.41**	233.84*	0.79**	0.77**	0.89**	88.51^{ns}

*, ** = significant at 5% and 1% probability, respectively, by the t–test. The variables highlighted in orange presented the highest significant values, while those highlighted in blue correspond to the significantly lower values. SD: Stem diameter; LA: Leaflet area; PH: Plant height; CLL: Central leaflet length; CLW: Central leaflet width; NPP: Number of pods per plant; GPL: Green pod length; NGG: Number of green grains, 100-GGW: 100-green-grain weight; GGY: green grain yield; DPL: dry pod length; NDG: Number of dry grains; 100-DGW: 100-dry-grains weight; DGY: dry grain yield.
