# Supplementary material for: Combining Ability and Hybrid Performance of Cowpea Landraces Under Semiarid Conditions
Source: Plants (Basel). 2026 Oct 14;15(20):3145. doi: 10.3390/plants15203145 (PMC13708615; doi:10.3390/plants15203145)
Supplement: Supplementary file 1 [file plants-15-03145-s001.zip › Table S4 - Supplementary Materials.pdf]

Supplementary Materials

Table S4. Comparison of 14 evaluated traits between parent lines and hybrids of cowpea (*Vigna unguiculata*) grown in a semiarid region of Northeast Brazil.

**Genotype**		**Traits**													
		**SD**	**LA**	**PH**	**CLL**	**CLW**	**NPP**	**GPL**	**NGG**	**100-GGW**	**GGY**	**DPL**	**NDG**	**100-DGW**	**DGY**
**Parents**	**L1**	1.51a	240.33a	100.66a	12.95a	9.36a	13.33b	21.87b	15.81a	21.93a	2576.29b	20.36b	15.55a	13.05a	1452.68b
	**L2**	1.29b	168.75a	96.22a	10.51b	7.80b	22.33b	19.61c	12.34b	12.01b	2244.35b	21.91a	14.00b	8.97b	1701.48b
	**L3**	1.35a	262.10a	77.38b	13.83a	8.88a	21.88b	25.13a	14.57a	21.87a	4032.96a	23.37a	13.77b	11.74a	2101.57a
	**L4**	1.01c	215.53a	24.88b	13.67a	8.25b	17.44b	19.42c	13.81b	11.96b	1731.94b	18.64b	13.44b	7.18b	1059.53b
	**L5**	1.22b	163.06b	66.55b	11.23b	7.88b	19.77b	22.48a	16.33a	20.72a	3402.03a	22.67a	16.22a	13.96a	2286.48a
	**L6**	1.51a	162.68b	99.77a	11.41b	7.96b	29.11a	19.26c	12.80b	15.51b	3734.62a	18.90b	14.11b	9.95b	2492.77a
**Hybrids**	**L1 x L2**	1.52a	225.30a	54.66b	12.36a	9.31a	23.55b	21.67b	15.55a	19.87a	4031.57a	21.27a	15.11a	12.76a	2607.12a
	**L1 x L3**	1.46a	231.11a	56.22b	12.84a	9.85a	23.11b	21.40b	13.26b	19.98a	3717.87a	21.72a	13.66b	13.36a	2717.87a
	**L1 x L4**	1.35a	179.99a	56.55b	11.45b	7.41b	34.77a	20.18c	14.52a	13.43b	3881.94a	20.77b	15.11a	10.27b	3003.42a
	**L1 x L5**	1.41a	210.29a	113.22a	10.82b	8.12b	18.33b	20.47c	15.33a	21.83a	3407.22a	19.12b	13.00b	9.44b	1528.24b
	**L1 x L6**	1.51a	210.03a	139.00a	11.81b	8.53a	27.33a	21.17b	14.76a	19.15a	4640.74a	19.98b	13.66b	9.56b	1766.20b
	**L2 x L3**	1.18b	228.95a	60.11b	12.23a	8.76a	26.11a	22.10b	13.55b	14.42b	2200.09b	19.05b	14.33b	8.44b	1257.59b
	**L2 x L4**	1.14c	298.28a	49.00b	11.74b	7.68b	17.77b	21.04b	13.81b	14.01b	3297.12a	20.64b	15.00a	8.78b	1974.90b
	**L2 x L5**	1.29b	199.91b	50.44b	12.00b	8.55a	27.44a	20.35c	14.75a	12.14b	2483.51b	20.38b	14.22b	9.54b	2204.25a
	**L2 x L6**	1.08c	175.16b	54.22b	11.27b	7.07b	17.55b	20.76b	13.81b	12.95b	1902.77b	19.57b	14.22b	8.33b	1242.03b
	**L3 x L4**	1.13c	218.95a	80.44a	12.83a	8.44b	20.33b	23.60a	14.36a	16.34b	2801.29b	21.86a	15.55b	10.27b	1738.51b
	**L3 x L5**	1.25b	228.48a	88.44a	13.74a	9.06a	20.00b	25.21a	15.43a	21.54a	3424.44a	23.12a	13.44b	11.83a	1942.95b
	**L3 x L6**	1.06c	217.01a	97.66a	12.92a	8.57a	24.33b	24.25a	15.93a	20.87a	3692.03a	23.14a	14.77a	10.94a	2311.94a
	**L4 x L5**	1.46a	236.86a	102.44a	13.01a	8.78a	25.55a	21.50b	13.85b	15.91b	3535.18a	21.67a	13.44b	11.17a	2425.54a
	**L4 x L6**	1.11c	196.62b	42.66b	12.48a	8.01b	19.11b	19.83c	15.32a	13.77b	2201.85b	21.05b	15.77a	10.03b	1579.07b
	**L5 x L6**	1.49a	210.14a	85.40a	11.78b	8.66a	17.88b	23.23a	15.80a	18.47a	2702.68b	20.55b	16.44a	12.93a	1893.98b

Table S3. Continuation…

**Reciprocal**	**L2 x L1**	1.28b	210.29a	97.0a	11.57b	8.33b	21.77b	20.11c	15.25a	16.36b	3240.46a	20.27b	15.00a	8.35b	1520.09b
	**L3 x L1**	1.24b	206.96a	44.44b	12.72a	8.55a	21.22b	22.17b	14.18b	17.65a	3256.20a	22.37a	14.33b	11.93a	2145.35a
	**L3 x L2**	1.19b	218.72a	54.00b	12.72a	8.88a	25.00a	21.55b	13.95b	15.93b	3360.18a	20.32b	13.00b	10.86a	2324.53a
	**L4 x L1**	1.36a	191.21a	82.11a	12.42a	8.07b	32.22a	19.70c	12.97b	12.39b	3127.22a	20.46b	14.77a	9.14b	2262.68a
	**L4 x L2**	0.98c	190,85b	58.33b	12.83a	8.18b	16.44b	18.43c	13.14b	13.15b	1772.68b	20.95b	14.66a	9.09b	1299.17b
	**L4 x L3**	1.29b	243.10a	74.22b	13.18a	8.37b	25.77a	21.17b	13.14b	14.48b	3238.42a	21.91a	14.00b	9.14b	1933.05b
	**L5 x L1**	1.44a	207.28a	82.22a	11.77b	8.58a	20.44b	22.16b	15.10a	22.93a	3839.44a	21.78a	16.55a	13.16a	2139.35a
	**L5 x L2**	1.32b	230.11a	84.22a	12.71a	9.16a	21.33b	21.87b	14.12b	15.74b	2931.20b	22.53a	14.77a	11.94a	2063.98a
	**L5 x L3**	1.58a	217.95a	100.88a	12.08b	8.56a	27.11a	22.27b	14.58a	17.12a	3878.79a	20.46b	15.11a	11.31a	2745.27a
	**L5 x L4**	1.52a	209.13a	76.00b	11.45b	8.08b	34.77a	20.93b	12.93b	15.48b	4515.00a	20.82b	14.33b	10.66a	3128.88a
	**L6 x L1**	1.69a	239.85a	110.22a	12.72a	9.17a	28.55a	20.55c	14.52a	17.87a	4255.55a	19.11b	15.11a	9.64b	2360.55a
	**L6 x L2**	1.26b	185.39b	111.28a	10.91b	7.38b	23.88b	21.20b	15.50a	16.63b	3126.38a	23.95a	14.11b	8.82b	1827.77b
	**L6 x L3**	1.42a	246.74a	106.11a	12.60a	8.14b	23.11b	25.44a	15.92a	21.86a	4187.96a	20.90b	16.55a	11.31a	2181.85a
	**L6 x L4**	1.23b	213.67a	58.77b	11.96b	8.18b	20.66b	19.94c	10.40b	14.10b	2215.83b	20.90b	14.88a	9.47b	1710.18b
	**L6 x L5**	1.61a	187.33b	143.33a	11.20b	8.11b	18.11b	21.26b	14.44a	15.65b	2235.00b	21.08b	14.88a	11.14a	1716.94b

Means followed by the same letter within a column do not differ significantly according to the Scott-Knott test at the 1% probability level. SD: Stem diameter; LA: Leaflet area; PH: Plant height; CLL: Central leaflet length; CLW: Central leaflet width; NPP: Number of pods per plant; GPL: Green pod length; NGG: Number of green grains, 100-GGW: 100-green-grain weight; GGY: green grain yield; DPL: dry pod length; NDG: Number of dry grains; 100-DGW: 100-dry-grains weight; DGY: dry grain yield.
