# Supplementary material for: Combining Ability and Hybrid Performance of Cowpea Landraces Under Semiarid Conditions
Source: Plants (Basel). 2026 Oct 14;15(20):3145. doi: 10.3390/plants15203145 (PMC13708615; doi:10.3390/plants15203145)
Supplement: Supplementary file 1 [file plants-15-03145-s001.zip › Table S5 - Supplementary Materials.pdf]

Supplementary Materials

Table S5. Climatic data recorded at the Experimental Farm of the Federal University of Paraíba (UFPB), located in the municipality of São João do Cariri, Paraíba, Brazil, during 2023.

**Months**	**Precipitation (mm)**	**Mean air temperature (°C)**	**Relative air humidity (%)**	**Mean soil temperature (°C) at 5 cm**
January	18.79	26.37	64.74	33.60
February	22.35	26.88	63.76	34.06
March	190.24	26.03	68.26	31.34
April	39.63	25.29	73.59	29.89
May	57.66	25.16	73.27	30.18
June	86.62	23.47	76.83	27.21
July	26.92	23.04	74.73	26.43
August	2.03	24.00	70.57	29.77
September	4.57	24.91	68.54	31.44
October	0.00	26.43	53.03	34.62
November	6.60	27.24	47.89	35.84
December	64.02	26.95	58.71	33.77

Data obtained from the meteorological station at the UFPB Experimental Farm in São João do Cariri, Paraíba, Brazil.
